# ﻿A cryptic new species of tiger swallowtail (Lepidoptera, Papilionidae) from eastern North America

**DOI:** 10.3897/zookeys.1228.142202

**Published:** 2025-02-14

**Authors:** Charles J. DeRoller, Xi Wang, Julian R. Dupuis, B. Christian Schmidt

**Affiliations:** 1 PO Box 374, Victor, New York, 14564, USA Unaffiliated New York United States of America; 2 Kingston, Ontario, Canada Unaffiliated Kingston Canada; 3 Department of Entomology, University of Kentucky, Lexington, KY, 40546, USA University of Kentucky Lexington United States of America; 4 Canadian National Collection of Insects, Arachnids, and Nematodes, Biodiversity Program, Agriculture and Agri-Food Canada, Ottawa, ON, Canada Canadian National Collection of Insects, Arachnids, and Nematodes, Biodiversity Program, Agriculture and Agri-Food Canada Ottawa Canada

**Keywords:** cryptic species, hybrid, *Papilioglaucus*, Papilionidae, *Pterourus*, speciation

## Abstract

In the eastern Great Lakes region of North America, two tiger swallowtail species have previously been recognized, *Papilioglaucus* Linnaeus, 1758 and *Papiliocanadensis* Rothschild & Jordan, 1906. A third entity, the Midsummer Tiger Swallowtail, has been treated as a P.glaucus×canadensis hybrid, and exhibits a mosaic of both intermediate and unique morphological and biological traits. Here we demonstrate that rather than being a localized, historically recent hybrid phenomenon, the Midsummer Tiger Swallowtail maintains its morphological and physiological distinctness over a large geographic region in the absence of one or both putative parental species, and was first documented in the literature nearly 150 years ago. *Papiliosolstitius***sp. nov.** is physiologically unique in delaying post-diapause development, which results in allochronic isolation between the spring flights of *P.glaucus* and *P.canadensis*, and the late summer flight of *P.glaucus*. Similarly, the geographic range of *Papiliosolstitius* spans the region between the northern terminus of *P.glaucus* and southern limits of *P.canadensis*, remaining distinct in areas of sympatry. Defining the taxonomic identity of this unique evolutionary lineage provides an important baseline for further inquiry into what has served as an exemplary species group in evolutionary study.

## ﻿Introduction

The North American *Papilioglaucus* species group (Lepidoptera: Papilionidae) is a model study system in insect evolutionary biology. The recognition and delimitation of *P.glaucus* L., 1758 and *P.canadensis* as a classic sibling species pair (Hagen et al. 1991; Sperling 1993) led to three decades of study in speciation, host plant adaptation, hybridization, and molecular evolution (e.g., Ryan et al. 2017 and references therein). More recently, the discovery of a third species, *P.appalachiensis* Pavulaan & Wright, 2002, has provided unprecedented insight into speciation via hybridization (Scriber and Ording 2005; Kunte et al. 2011; Cong et al. 2015; Vernygora et al. 2022). *Papilioappalachiensis* is now recognized as a homoploid hybrid species with origins from *P.glaucus* × *P.canadensis* crosses some 0.4 million years ago (Cong et al. 2015; Kunte et al. 2011).

The *Papilioglaucus* group previously comprised nine species (Kunte et al. 2011; Pavulaan 2024), and the five eastern North American species discussed herein are termed the *glaucus* complex. All are very similar in external appearance, and prior to 1991, were included within the concept of a single species, *P.glaucus*. Subsequently, three species were recognized: The Eastern Tiger Swallowtail (*P.glaucus*) which occurs across most of eastern USA and as far north as southwestern Ontario, and south of the Adirondack and Catskill Mountains in New York; the more northern Canadian Tiger Swallowtail (*P.canadensis*) that occurs across the boreal region from Newfoundland to Alaska, and as far south as southern Ontario and the northern Appalachians; and the Appalachian Mountains endemic *P.appalachiensis*, found from Pennsylvania to Georgia (Pavulaan and Wright 2002). The recently described New England Swallowtail, *Papiliobjorkae* Pavulaan, 2024, may be conspecific with *P.canadensis* or *P.glaucus*; as detailed below in “Comparative morphology of the *Papilioglaucus*-complex,” incomplete knowledge of *P.bjorkae*’s morphology, range, biology, and taxonomic status currently precludes full comparison to the remainder of the *P.glaucus* group.

The *Papilioglaucus* group is part of a larger, predominantly New World clade of swallowtails of the subgenus Pterourus Scopoli, sometimes recognized as a distinct genus (e.g., Pelham and Pohl 2023). The broader concept of the genus *Papilio* L. is used herein, in agreement with the results and reasoning presented by Condamine et al. (2023).

**Figure 1.**
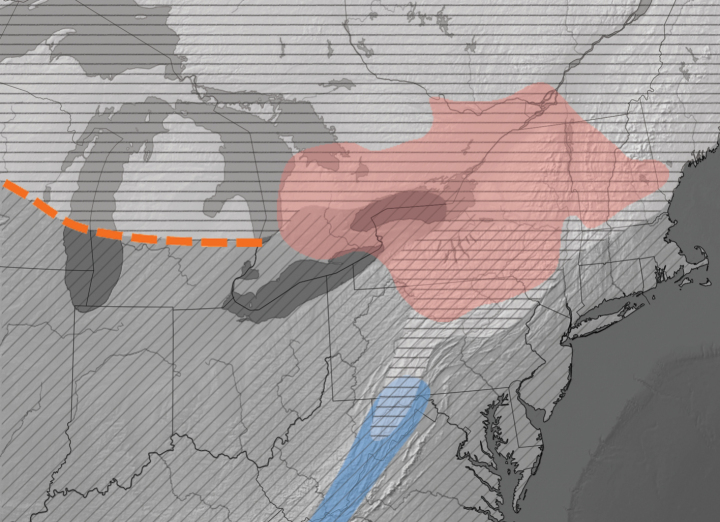
Geographic ranges of the *Papilioglaucus*-complex in eastern North America. *Papilioglaucus* (diagonal lines), *P.canadensis* (horizontal lines), *P.appalachiensis* (blue), and *P.solstitius* sp. nov. (red). In the central Great Lakes region, a sharp transition or hybrid zone occurs between *P.glaucus* to the south and *P.canadensis* to the north, indicated by the orange dashed line. In the northern Appalachian region this transition zone is much larger as a result of topography-induced climatic variation, with elevational rather than latitudinal separation. Considerable uncertainty exists in the northern range limit of *P.glaucus* in NY (see “Habitat and distribution” section). Distribution data based on Luebke et al. (1988); Stump et al. (2003); Pavulaan and Wright (2002); Mcnaughton et al. (2020) and specimens verified in this study (Suppl. material 1).

Each of the *glaucus*-complex species show adaptation to different thermal niches that can be broadly characterized as warm (*P.glaucus*), intermediate (*P.appalachiensis*), and cool (*P.canadensis*) climatic regions; all have broad larval host plant diets, and are not restricted by the distributions thereof. At coarse geographic scales, species distributions appear parapatric, but at finer spatial scales, multiple taxa can overlap (Fig. 1). The transition or contact zone between *P.glaucus* and *P.canadensis* has received considerable study. West of Lake Michigan, introgression and hybridization have been well-documented through morphometric and molecular studies (Luebke et al. 1988; Ryan et al. 2016, 2017, 2018). Here, a narrow hybrid zone (50–100 km wide) is maintained by strong selective pressure for adaptation to either warm or cool thermal regimes, with a rapid geographic shift from *P.glaucus* to *P.canadensis* across a threshold thermocline (Fig. 1; Scriber 2010; Ryan et al. 2016, 2017, 2018).

In the topographically and climatically complex region of eastern Ontario and adjacent New York, the relationship between *P.canadensis* and *P.glaucus* is less straightforward. Unlike the central Great Lakes region to the west, the ranges of *P.canadensis* and *P.glaucus* are more poorly defined as a result of confusing phenotypes and phenologies, making identification difficult. In northern New York, Vermont and eastern Ontario, univoltine tiger swallowtails with a July flight period have variously been called *P.glaucus* (Shapiro 1974; Layberry et al. 1998; Hall et al. 2014), “false second generation” (Hagen and Lederhouse 1985), “late flight *P.canadensis*” (Scriber and Ording 2005; Kunte et al. 2011), “hybrid types” (Scriber 1990), “late flight” (Scriber 2010), “late flight hybrids” (Wang 2018), “delayed ‘late flight’ hybrid swarm” (Scriber 2010), “a stable hybrid” (Zhang et al. 2013), “intermediate individuals” (Vernygora et al. 2022), “midsummer tiger swallowtail” (Schmidt 2020), and “divergent ecomorphs” (Vernygora et al. 2022). Hagen and Lederhouse proved that this taxon is not the second annual generation of any spring-flying swallowtails, instead representing a single-brooded taxon physiologically distinct from *P.glaucus* and *P.canadensis* (Hagen and Lederhouse 1984; Scriber and Ording 2005). This taxon is now referred to by the common name Midsummer Tiger Swallowtail (MST; Schmidt 2020).

Here, we present evidence that MST is not the result of historically recent hybridization between *P.glaucus* and *P.canadensis* as suggested by Kunte et al. (2011); literature and specimen records of MST date back 150 and 50 years, respectively. MST was also previously thought to be geographically localized to areas of *P.glaucus* - *P.canadensis* overlap, but this is also not the case. MST exhibits a large geographic range that includes regions where one or even both putative parent species are absent (Fig. 1). Lastly, the unique late-season flight acts as an allochronic reproductive barrier between MST and other tiger swallowtails. Based on combined molecular, phenological, morphological, and natural history data, the Midsummer Tiger Swallowtail is described as a new species, *Papiliosolstitius* sp. nov.

### ﻿Methods and materials

Field studies and specimen collections were carried out from 1999 to 2023 in Pennsylvania, Virginia, Kentucky, and the Finger Lakes region of New York (CJD); and from 2008 to 2023 in eastern Ontario (XW, BCS). Host plant suitability, larval development, and adult emergence were studied based on *ex ova* and *ex larva* rearings from 2008 to 2011 in Hamilton, Ontario, and from 2015 to 2022 in Kingston, Ontario (XW). All larvae were reared indoors at a constant 23 °C under outdoor ambient light conditions. Larvae were provided with cuttings of the host they were found on, either green ash (*Fraxinuspennsylvanicus* Marshall) or black cherry (*Prunusserotina* Ehrhart), held in small vials of water. Pupae that were entering diapause rather than direct development did not exhibit melanization of the eyes (visible by transillumination) after 2–3 weeks and were placed in cold storage, either in a conventional refrigerator or unheated garage. After removal from cold storage, they were again kept at a constant 23 °C and time to eclosion recorded. Adult genitalia were prepared following the protocol detailed in Schmidt (2018) and imaged using a Leica DFC 450 camera mounted on a Leica M205C stereo microscope.

Where confident identification was possible, distribution and phenology data were augmented with records from iNaturalist (inaturalist.org), eButterfly (e-butterfly.org), and the Ontario Butterfly Atlas (Macnaughton et al. 2020). Manual calipers precise to the nearest 0.1 mm were used for wing measurements. Occurrence maps were created with SimpleMappr (https://www.simplemappr.net). Voucher specimens examined in this study (Suppl. material 1) are found in the following collections:

**CNC** Canadian National Collection of Insects, Arachnids and Nematodes, Ottawa, CAN

**CMNH** Carnegie Museum of Natural History, Pittsburgh, PA, USA

**CJDC** Charles J. DeRoller Collection

**XWC** Xi Wang Collection

### ﻿Molecular datasets

Publicly available sequences and previous DNA barcoding efforts in the *P.glaucus* group have focused on both the 5’ region of the mitochondrial *cytochrome oxidase subunit I* (COI) gene (the standard barcode region (Hebert et al. 2003) using primers LCO1490 and HCO2198 (Folmer et al. 1994)) and the 3’ region of COI (primers Jerry and Pat, as used in Kunte et al. 2011; Vernygora et al. 2022). Unfortunately, few, if any, specimens have been sequenced for both regions, so we are limited to considering these regions separately and hereafter refer to them as COI5 and COI3, respectively. Fourteen MST specimens were sent to a private COI5 barcoding service; Sanger sequencing was performed by Azenta Life Sciences (Chelmsford, Massachusetts, United States), and consensus sequences were constructed using de novo assembly in Geneious Prime v. 2024.0 (uploaded to BOLD with accessions provided in the associated figure). Additionally, COI5 barcodes were generated for two *P.appalachiensis* (UASM400650 and UASM400651, also sequenced by Vernygora et al. 2022), to ensure representation of that species in the COI5 dataset (NCBI GenBank accessions: PQ578215.1 and PQ578216.1). Sequencing and analysis were conducted as in Vernygora et al. (2022). COI5 and COI3 sequences were retrieved from GenBank (September 2024) for all species in the *glaucus*-complex and aligned to a complete *P.glaucus* mitogenome (NC_027252.1). Outgroup taxa were also selected as in Vernygora et al. (2022). Unique and pertinent COI5 sequences in the BOLD database (i.e., those of *P.glaucus* and *P.canadensis* from NE USA and SE Canada) were added to this dataset, and we used AliView v1.28 (Larsson 2014) to align sequences either manually or using default settings with MUSCLE (Edgar 2004). We used IQ-Tree v. 2.3.5 (Nguyen et al. 2015) to conduct maximum likelihood tree searches using the best model identified by Bayesian Information Criterion with ModelFinder (Kalyaanamoorthy et al. 2017). One thousand replicates of ultra-fast bootstrap (ufBS, Hoang et al. 2018) and the Shimodaira-Hasegawa approximate likelihood ratio test (SH-aLRT, Guindon et al. 2010) were used to assess nodal support. The genomic phylogeny using 3,733 single nucleotide polymorphisms (SNPs) from Vernygora et al. (2022) was also considered (we focused on the majority rule consensus tree generated from MrBayes (Ronquist et al. 2012), although see Vernygora et al. (2022) for more details on their thorough analysis), and we reevaluated the morphology of those specimens noted as “intermediates” in their analyses. All trees were visualized with FigTree v. 1.4.4 (Rambaut and Drummond 2010).

## ﻿Results

Taxonomic names currently in synonymy under *P.glaucus* and *P.canadensis* were reviewed and revised by Pavulaan and Wright (2002). Our review of these synonymies confirms that all taxon names are correctly attributed to their respective species, and do not apply to the Midsummer Tiger Swallowtail. As such, a new name is proposed here.

### ﻿ Papilio solstitius sp. nov.

Taxon classificationAnimaliaLepidopteraPapilionidae

384185E1-BC5E-586F-9B6E-8AB233D37B4A

https://zoobank.org/A9B99C5C-E8EC-4AA1-A6E6-B09E610E3389

Figs 3a , 4 , 5 , 6a , 7a , 8a , 9c-d , 10a , 11

#### Type locality.

Canada, Ontario, Ottawa-Carleton District, Long Swamp, Old Almonte Rd., 45.249°N, 76.079°W.

#### Type material.

***Holotype*** (Fig. 4a) • male. Ontario, Ottawa-Carleton Dist., Old Almonte Rd. at Long Swamp, 45.249°N, 76.079°W, 3.Jul.2020, B.C. Schmidt, CNC voucher # CNCLEP00342771 [CNC]. ***Allotype*** (Fig. 4b) • female. Ontario, Frontenac Co., Vanalstine Lake, 44.858°N, 76.847°W, 5.Jul.2021, B. C. Schmidt, observed ovipositing on *Prunusserotina* [CNC]. ***Paratypes*** • 53 in CNC, 9 in XWC, 8 in CJDC; complete data and specimen deposition are given in Suppl. material 1.

#### Etymology.

The epithet solstitius is derived from solstitium, the Latin term for solstice. The species’ unique midsummer flight period commences near the summer solstice.

#### Differential diagnosis.

**Figure 2.**
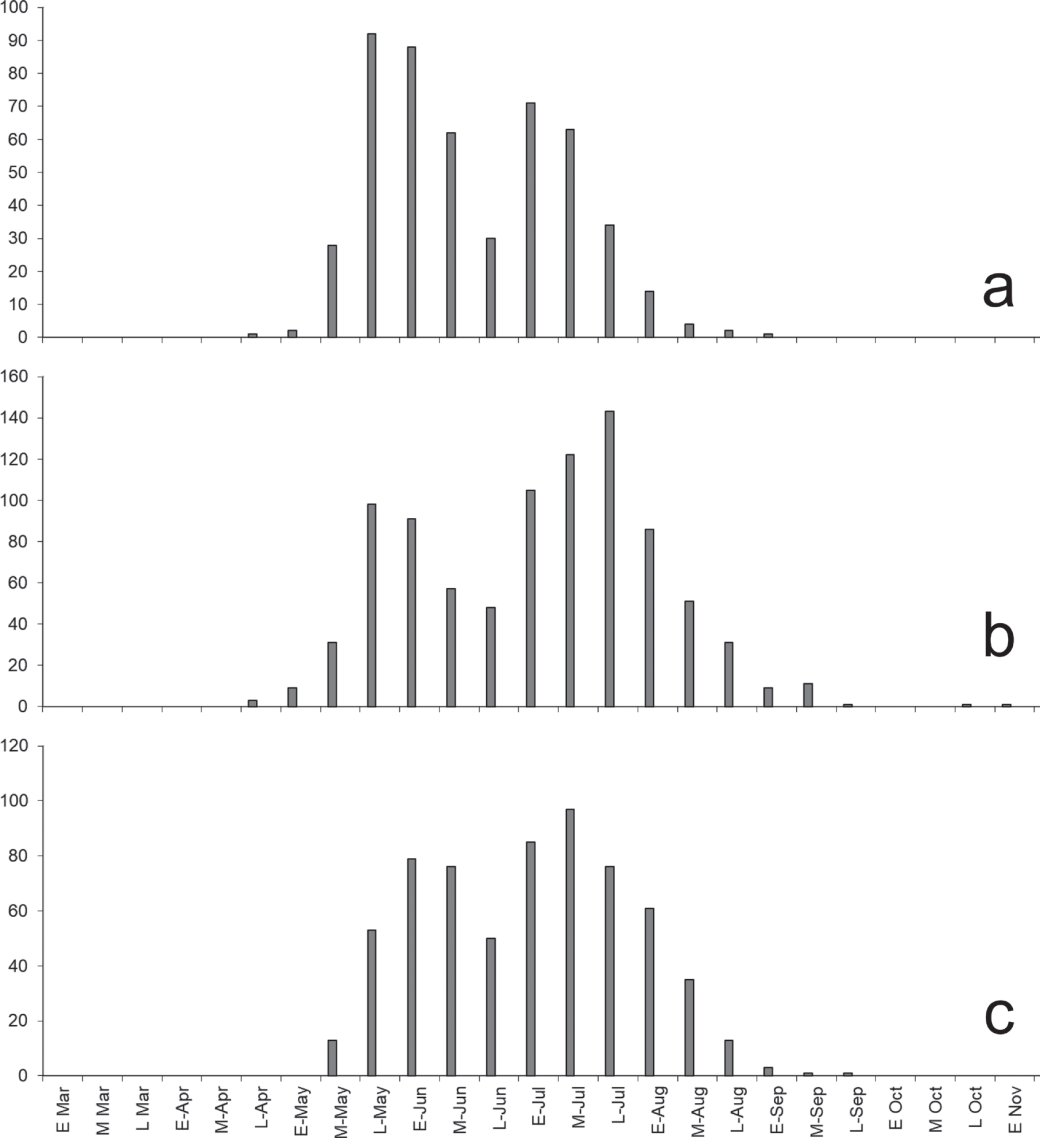
Phenology of *Papilioglaucus* group species from three regions, based on combined observations for all species and grouped by 10-day intervals **a** eastern Ontario (Hastings Co., Frontenac Co.; Mcnaughton et al. (2020)), with *P.canadensis* peaking in late May – early June and *P.solstitius* in early to mid-July **b** finger Lakes region, New York (iNaturalist), with a late May – early June peak of spring *P.glaucus* and a July – August peak of *P.solstitius* and summer *P.glaucus*; note later seasonal persistence and late-shifted peak resulting from summer *P.glaucus*, which is absent in eastern Ontario (2a) **c** Greater Toronto region, with a spring peak comprised of *P.canadensis* and spring brood *P.glaucus*, and a July peak of *P.solstitius*; summer brood (August) *P.glaucus* are very rare.

*Papiliosolstitius* is closely related to *P.glaucus*, *P.canadensis* and *P.appalachiensis*, but differs from all in a suite of characters (Table 1). The most significant differences are apparent in developmental biology and phenology. *Papiliosolstitius* is unique in its long post-diapause emergence delay, with adult eclosion beginning in late June to early July, compared to May for all other species (Fig. 2). Unlike the facultatively multivoltine *P.glaucus*, *P.solstitius* is obligately univoltine (like *P.canadensis* and *P.appalachiensis*). In the northern part of its range, *P.solstitius* overlaps with *P.canadensis*, and in the south with *P.glaucus*; it is not known to overlap with *P.appalachiensis* (Fig. 1). Identification difficulties are therefore largely limited to confusion with either *P.canadensis* or *P.glaucus*. In combination with location and date, the comparative morphological characters summarized in Table 1 and discussed in the “Comparative Morphology” section below will serve to identify most specimens.

**Table 1.** Comparison of morphological traits among species of the *Papilioglaucus*-complex. FW = forewing; DFW = dorsal forewing; VFW = ventral forewing; HW = hindwing; DHW = dorsal hindwing; VHW = ventral hindwing. Forewing length is geographically variable in *P.canadensis* and *P.glaucus*, and values are based on Ontario specimens.

Trait	*P.solstitius* sp. nov.	*P.canadensis*	*P.glaucus*	*P.appalachiensis*	P.bjorkae*
**Head - setation of frons**	intermediate, compact	long and diffuse	short and compact	intermediate, compact	short and compact
**Average FW length (range): male**	51 mm (42–57 mm)	46 mm (41–50 mm)	spring: 50 mm (43–52 mm); summer: 54 mm (45–58 mm)	50–62 mm	male and female combined: 49.2 mm (43–55 mm)
**Average FW length (range): female**	53 mm (48–56 mm)	48 mm (47–50 mm)	spring: 53 mm (50–55 mm); summer: 57 (49–64 mm)	50–65 mm	(unknown)
**FW shape - distal margin**	usually straight to slightly concave; concave frequency 40–50%	usually straight to slightly convex; concave frequency 25–30%	usually concave; concave frequency >80%	usually straight	concave
**DFW - frequency of medial band black scales extending beyond Cu2 (male)**	10–15%	55–70%	< 15%	< 20%	(unknown)
**VFW margin: submarginal band**	broadly coalescent lunules, usually with scalloped inner border	continuous band with straight inner and outer border; varying to coalescent rounded-rectangular elements, but lunules never well-separated by black	discrete lunules distinctly separated by black line along veins; varying to coalesced lunules with scalloped inner and outer margin	broadly coalescent lunules, usually with scalloped inner border	continuous band, sometimes with coalescent lunules anteriorly
**VFW margin: inner (proximal) border**	moderate amount of yellow dusting over black inner half	extensive yellow dusting over black inner half	extensive yellow dusting over black inner half	extensive yellow dusting over black inner half	extensive yellow dusting over black inner half
**HW shape**	elongate	broad / rounded	elongate	more triangular than *glaucus*	more angular than *glaucus*
**HW tails**	spatulate	aspatulate to slightly spatulate	spatulate	aspatulate to slightly spatulate	slightly to well-spatulate
**HW margin**	less scalloped	less scalloped	scalloped	less scalloped	less scalloped
**HW anal cell black band width (male)**	40–50%	55–90%	10–40% (summer); 20–50% (spring)	average ~50%	40–50% (based on 2 illustrated specimens)
**DHW (female) submarginal orange lunule in cell Sc+R1**	Smaller than remaining lunules, sometimes a mere dot	Smaller than remaining lunules, sometimes a mere dot	Much larger than remaining lunules	Slightly larger than remaining lunules	Slightly larger than remaining lunules
**DHW female blue scaling**	sparse	none to minimal	sparse to extensive	sparse	sparse
**VHW marginal lunules**	lunules rectangular to slightly crescentic	lunules more rectangular	crescentic lunules	lunules more rectangular	lunules rectangular to slightly crescentic
**VHW marginal lunule ScR1 of female**	length less than that of other lunules, often much more so	length less than that of other lunules, often much more so	conspicuously larger/deeper than other lunules	similar in size to other lunules	conspicuously larger/deeper than other lunules
**VHW submarginal black band: inner border of 3 interspaces between Sc to M2**	slightly scalloped	more linear than scalloped	scalloped	slightly scalloped	scalloped
**VHW anal margin setation**	sparse setation	long, dense setation	sparse to very sparse setation	sparse setation	(not given)
**Abdomen shape**	narrow, attenuated anteriorly	shorter, broad anteriorly	narrow, attenuated anteriorly	moderately attenuated anteriorly	(not given)
**Abdomen subdorsal yellow stripe**	broad, bright yellow, lateral black line well defined but narrow	narrower, less vivid yellow; sublateral black line wide	broad, bright yellow, sublateral black line faint or partially absent	broad, bright yellow, lateral black line well defined but narrow	(not given)
**Male valve scales**	solid yellow scales; clasper same shade abdomen	yellow with sparse black scales, clasper often appearing darker than abdomen	solid yellow scales; clasper same shade abdomen	solid yellow scales; clasper same shade abdomen	(not given)
**Larva: 1^st^ instar posterior white patch**	usually present; rarely absent or faint	always present and well-developed	absent	absent or faint (tan)	unknown
**Larva: 1^st^ instar anterior white patch**	usually present; rarely absent or faint	always present and well-developed	absent	absent	unknown

* based on images and information in Pavulaan (2024a).

#### Description of adult.

**Figure 3.**
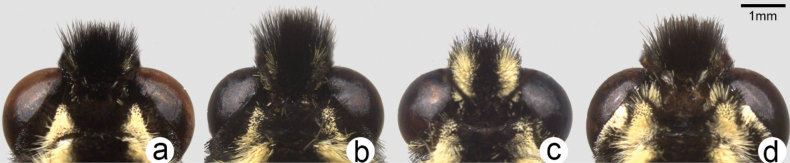
Dorsal view of head (antennae removed for clarity) comparing profile of frontal setae in **a***P.solstitius***b***P.canadensis***c***P.glaucus* and **d***P.appalachiensis*. Scale bar: 1 mm.

**Figure 4.**
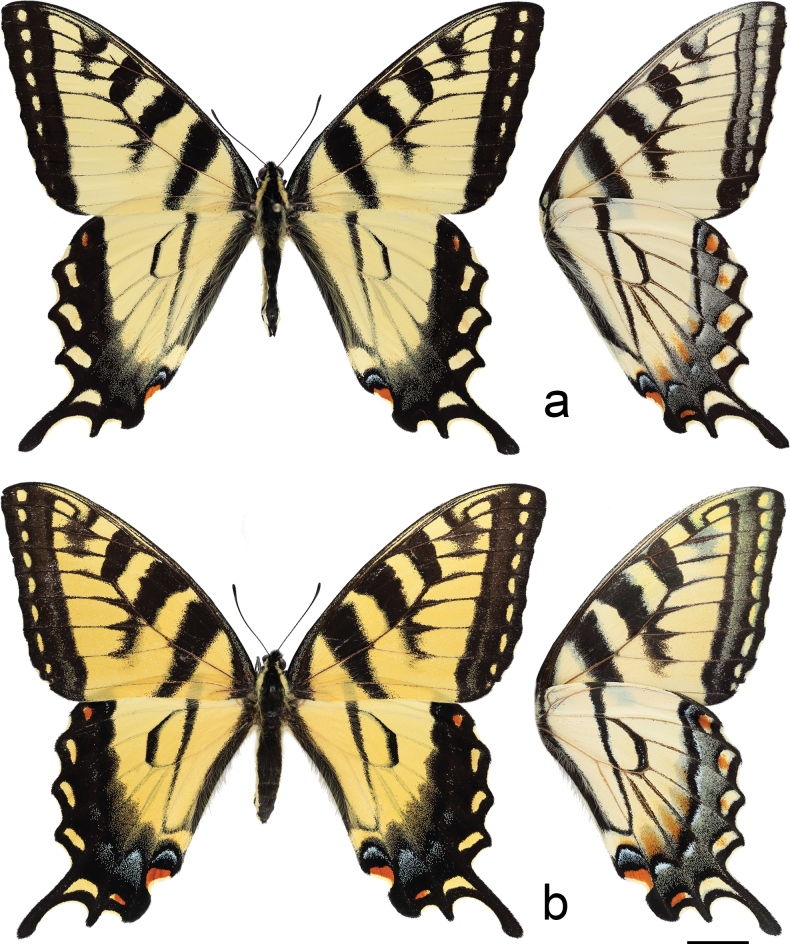
**a** dorsum of *Papiliosolstitius*, male, holotype, ventrum on right. Long Swamp, Old Almonte Rd., Ottawa, Ontario, CAN. CNC voucher # CNCLEP00342771 **b** dorsum of *Papiliosolstitius*, female allotype, ventrum on right. Vanalstine Lake, Frontenac Co., Ontario, CAN; ovipositing on *Prunusserotina*. Scale bar:10 mm.

**Figure 5.**
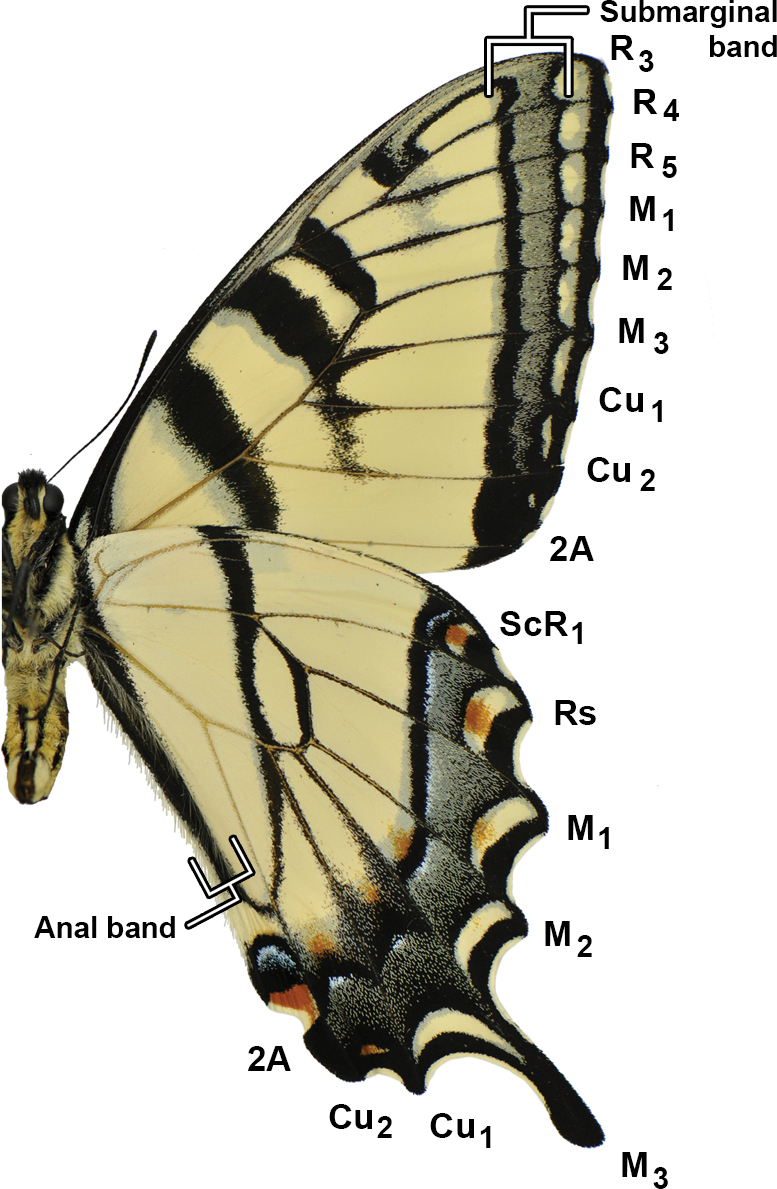
*Papiliosolstitius*, ventrum, wing vein and pattern terminology.

**Figure 6.**
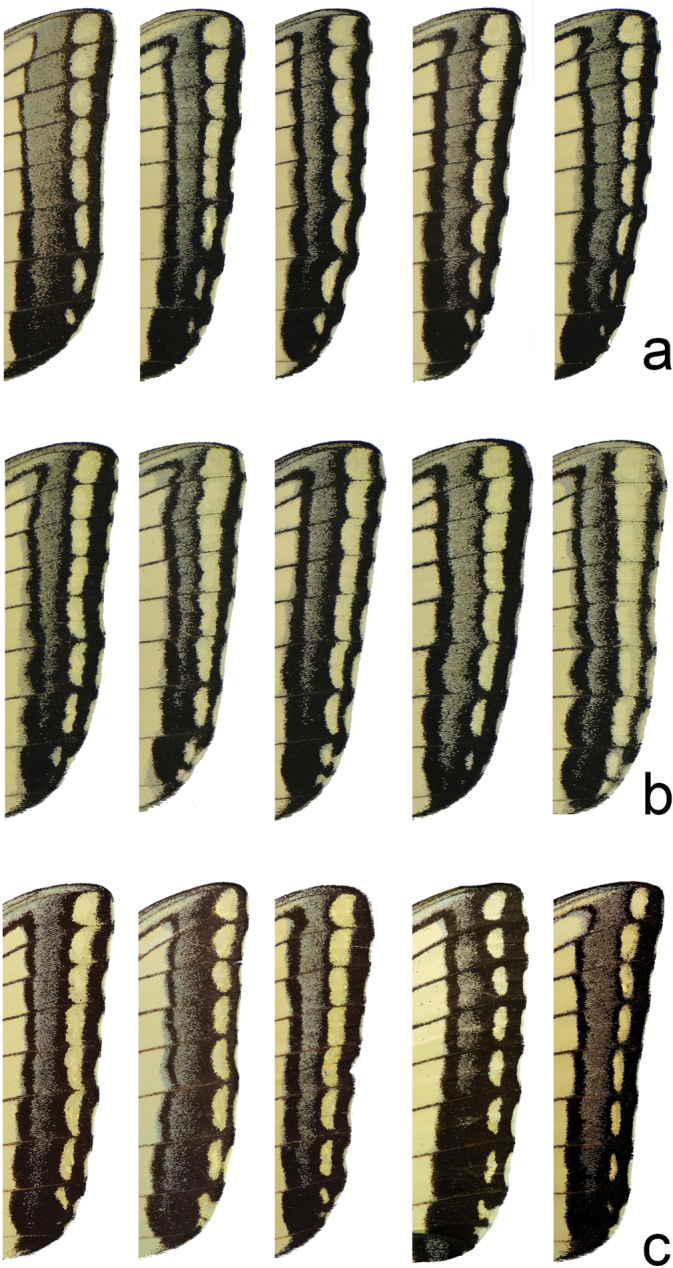
Comparison of variation in ventral forewing submarginal band in **a***P.solstitius***b***P.canadensis* and **c***P.glaucus*.

**Figure 7.**
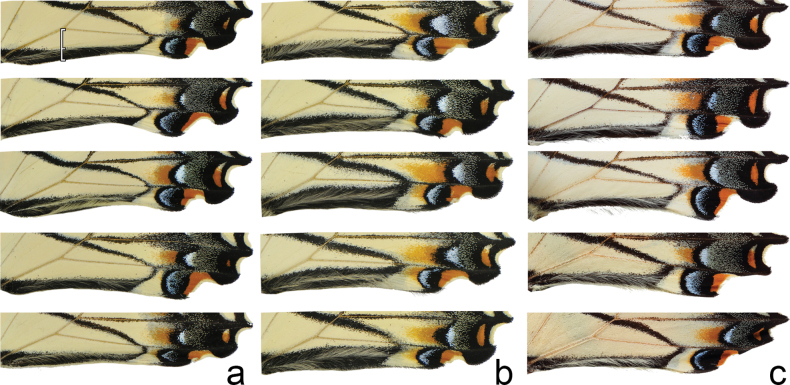
Comparison of variation in ventral hindwing anal band in **a***P.solstitius* and **b***P.canadensis* and **c***P.glaucus*.

**Figure 8.**
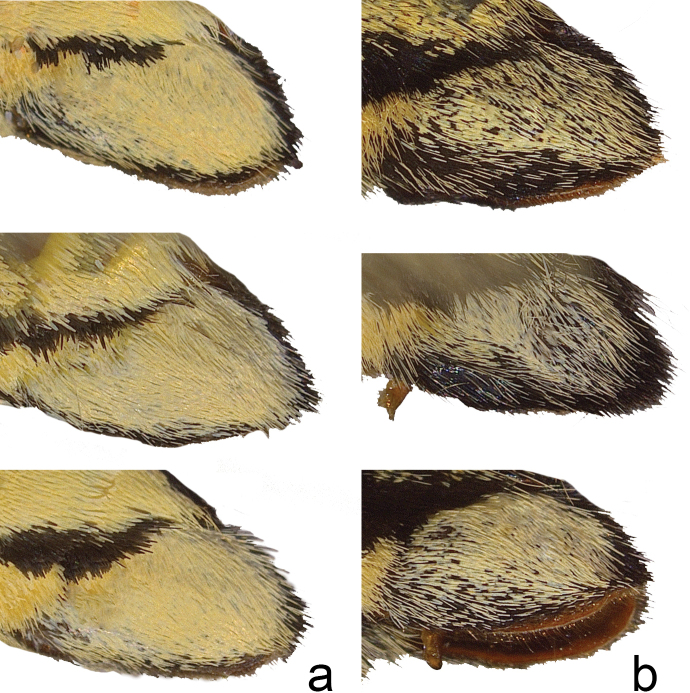
Comparison of scale coloration of male valve in **a***P.solstitius* and **b***P.canadensis*. Clasper color in *P.glaucus* (not shown) is identical to *P.solstitius*

**Figure 9.**
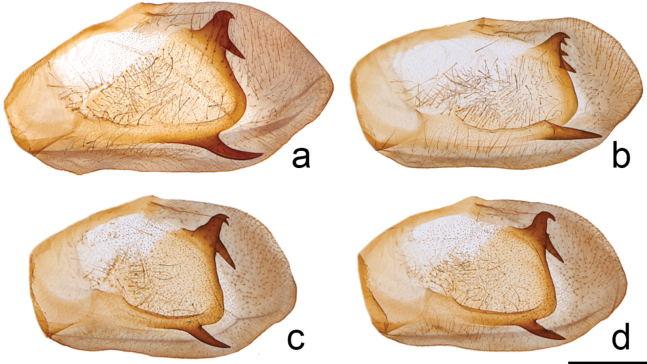
Inner surface of male right valve of **a***P.solstitius***b***P.glaucus***c, d***P.canadensis***c** and **d** show variation in dorsal clasper tines from the same individual (image of left valve (**d**) is flipped for ease of comparison).

Head (Fig. 3) and thorax: setation of frons of moderate length, intermediate between *P.canadensis* and *P.glaucus*; dorsum of head and thorax with limited sublateral yellow scaling; ventral thorax vestiture pale lemon yellow, legs black. Forewing (Figs 4, 5, 6): Male forewing length 50.7 mm (46.7–55.0 mm; *n* = 17), female 53.4 mm (47.7–57.0 mm; *n* = 8); dorsal ground color of male mustard yellow (Ridgway 1912), of female light orange yellow (Ridgway 1912), like that of *P.glaucus* but slightly richer in tone than *P.canadensis*; female mimetic dark phase absent; all pattern elements flat black; antemedial band an elongate wedge variable in thickness and edge, on average attenuating more strongly between Cu and anal margin than in *P.canadensis*; medial band an irregular rectangular bar across discal cell, variably extending as far as vein Cu_2_ or slightly beyond (in *P.canadensis* the medial band is more extensive, more frequently extending past Cu_2_ and sometimes to 2A); subapical black bar well-defined in cell R_3_-R_4_, diminishing across R_5_-M_1_, more strongly so than in *P.canadensis*; costa and subapical bar with diffuse yellow streaking, generally more so than in *P.canadensis*; females with wider, more diffuse transverse black bands than males; marginal band solid black with 6–8 yellow rounded-ovoid submarginal spots in interspaces; pattern elements repeated on ventral forewing, but ground color paler yellow, and black elements of distal half of wing with a flush of yellow scales; submarginal band variable but comprised of essentially D-shaped yellow spots usually separated by black lines along veins; yellow spots wider and more confluent than in *P.glaucus*, but more discrete and irregular than the essentially continuous, even-bordered band of *P.canadensis*. Hindwing: (Figs 4, 5, 7): Like *P.glaucus*, the scalloping of the hindwing outer margin is more pronounced than in *P.canadensis*, as a result of the disc margins oriented closer to the perpendicular of the long axis of the hindwing; the tail and Cu_2_ angle are slightly more lunate/lobate than in *P.canadensis*; ground color identical to that of forewing; inner margin bordered in black across 35–50% of cell 2A-Cu_2_; narrow, straight medial line attenuating towards juncture with anal band near Cu_2_; end of discal cell veins black-scaled; black marginal band extending along distal quarter of wing, with diffuse yellow dusting from vein M_2_ to anal angle; yellow submarginal lunules in the four cell spaces between Rs and Cu1; lunules of cell ScR_1_-Rs and Cu_2_-Cu_1_ (i.e., the uppermost and lowermost lunules) reduced or absent, orange or orange and yellow when present; anal angle with orange crescent capped proximally with blue, black bordered crescent; males with diffuse blue crescent in cell Cu_1_-Cu_2_, often faint, rarely traces of blue crescent in adjacent cell Cu_1_-M_3_; females with more extensive blue scaling, often with diffuse crescents extending to costal edge of submarginal band; ventral hindwing paler than dorsum, and with dusting of yellow scales across marginal band, and with more prevalent orange scaling in submarginal lunules and basad of marginal band in cells M_3_-2A; yellow setae along anal band shorter and sparser than in *P.canadensis*. Abdomen: dorsum black, pale yellow laterally and ventrally with black sublateral line; vestiture of mixed yellow and black fine, setae; scales of male clasper entirely yellow (Fig. 8); clasper of male valve with two dorsal tines (Fig. 9).

#### Description of larva.

**Figure 10.**
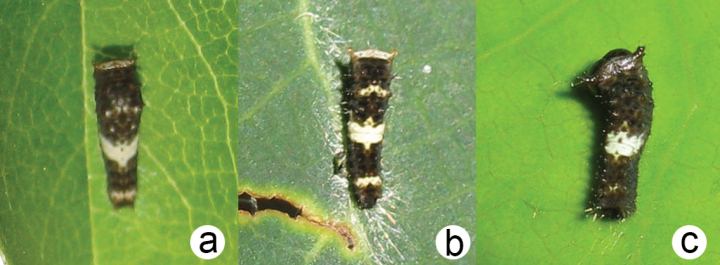
First instar of **a***P.solstitius***b***P.canadensis* and **c***P.glaucus*

**Figure 11.**
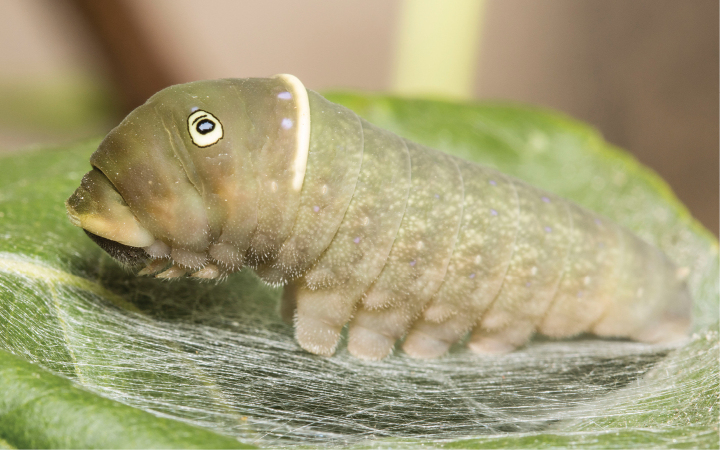
Mature larva of *P.solstitius* on hop-tree (*Pteleatrifoliata*), Ottawa, Ontario, CAN (H. Goulet, photograph).

First instar (Fig. 10) with well-developed white medial saddle, comprised of predominantly white dorsal pigmentation of segments A3-A4; three additional, variably developed white bands, one each comprised of T1 and T3, and a posterior band formed by A8; Anterior and posterior bands rarely absent (entirely brown pigmentation); mature larva (Fig. 11) indistinguishable from that of *P.glaucus* and *P.canadensis*.

##### ﻿Comparative morphology of the *Papilioglaucus*-complex

Adult morphology of all eastern North American species in the *glaucus*-complex can be deceivingly similar, and any single morphological character should not be relied upon for identification. Most similar to *P.solstitius* are *P.glaucus*, *P.canadensis* and potentially *P.bjorkae*, another new species in the *glaucus*-complex proposed in 2024 (Pavulaan 2024). Given its recency, the taxonomic status of *P.bjorkae* has not yet been scrutinized by the scientific community, but it is necessary to do so here. For the reasons detailed below the recognition and diagnosis of *P.bjorkae* is currently problematic, although based on the spring flight period and comparison of the figures in the original description (Pavulaan 2024), it is certain the name does not apply to MST.

The justification for treating *P.bjorkae* as a distinct species hinges on recognition of three distinct, partially sympatric, spring-flying taxa, recognized by adult phenotypes (*P.glaucus*, P.“nearcanadensis,” *P.bjorkae*) which correlate with slightly different flight periods (Pavulaan 2024). No diagnostic differences in immature stages, biology, larval hosts, or molecular markers of *P.bjorkae* have been documented to date (Pavulaan 2024), nor is there evidence in previous research that might hint at the existence of such (e.g., Ording et al. 2010; Kunte et al. 2011). Using seasonal adult abundance peaks combined across the *glaucus*-complex, flight phenologies for taxa present within the range of *P.bjorkae* are attributed to spring (*P.glaucus*, *P.canadensis*, and *P.bjorkae*), summer (midsummer swallowtail), and late summer (second-generation *P.glaucus*) (Pavulaan 2024: figs 3–5). During spring (May through June), *P.bjorkae* flies in “late spring,” versus “early spring” for *P.glaucus* and *P.canadensis*. However, only a single spring abundance peak is evident and attributed to *P.bjorkae*, whereas neither *P.glaucus* nor *P.canadensis* peaks are distinguishable due to the relative scarcity of observations for these species (Pavulaan 2024: 7, figs 3, 4). No additional data are provided to define late- versus early spring, leaving it unclear to what extent the phenology of *P.bjorkae* differs. Life history data that could corroborate such a difference are currently lacking.

The differential diagnosis of *P.bjorkae* is based largely on differences in wing pattern and shape, especially of the female (Table 1). Males are described as intermediate between *P.glaucus* and *P.canadensis*; comparative differences are given compared to *P.appalachiensis* and *P.canadensis*, but not *P.glaucus* (Pavulaan 2024: 16). Without an indication of sample size and a full description of male and female morphology, it is currently difficult to gauge intra- versus interspecific variation. Lastly, *P.bjorkae* is stated to be larger than spring *P.glaucus* and *P.canadensis*, but conflicting information on p. 9 states that *P.glaucus* is the largest species in the study region. No size measurements specific to male or female are given for *P.bjorkae* (including the holotype), nor is it possible to infer size of specimens from figures since scale bars are not given; size as a diagnostic trait for *P.bjorkae* therefore remains undefined.

The adult phenotype of *P.bjorkae* is very similar to that of *P.canadensis* and *P.glaucus*, so attributing phenotypic variation to three different putative taxa requires careful assessment. A potential additional source of phenotypic variation which remains unstudied stems from seasonal polymorphism in *P.glaucus*. Contrary to the assumption that *P.glaucus* is obligately bivoltine at the northern range edge (Pavulaan 2024), Ryan et al. (2016) demonstrate that it can be uni- or bivoltine depending on thermal constraints. In other words, temperature and day length experienced during the larval stage of *P.glaucus* dictate whether or not pupae develop directly into second generation adults, or enter winter diapause to emerge the following spring (Ryan et al. 2016). Since adult phenotype of *P.glaucus* is influenced by different temperature-photoperiod profiles (different spring and summer forms are well-known in *P.glaucus*, e.g., Pavulaan and Wright 2002), populations that comprise uni- and bivoltine cohorts would be expected to exhibit bimodal spring phenotypes (i.e., those developed from previous year’s spring versus summer adults). If proven, phenotypic variation driven by facultative voltinism in *P.glaucus* could account for the perception of phenotypes that are unaccounted for with existing taxonomy.

It is evident that the descriptive and diagnostic information defining *P.bjorkae* is currently incomplete and partially contradictory, and corroborating evidence for its distinctness as a species, outside of adult morphology, is lacking. This renders the recognition of *P.bjorkae* as a valid species tenuous at best. To spur further inquiry and study, we nevertheless include the known comparative phenotypic traits in Table 1.

Despite the overall similarity of *P.solstitius* to *P.glaucus*, we have found that it is possible to confidently identify the vast majority of individuals when multiple diagnostic traits are assessed. *Papiliosolstitius* is most similar to the northernmost populations of spring generation *P.glaucus*, and some specimens are not distinguishable based on wing pattern alone. *Papiliosolstitius* differs from *P.glaucus* in smaller overall size, greater tendency for the ventral forewing submarginal band to be band-like (broken into rounded crescents interrupted by black veins in typical *P.glaucus*); less scalloped outer border of the ventral hindwing submarginal band, and the absence of dark phase females (present in both *P.glaucus* and *P.appalachiensis*). The forewing outer margin is less frequently concave than in *P.glaucus*. Variation in these wing pattern traits often overlap with those of *P.glaucus*, and specimen identification requires consideration of seasonal timing and location. In *P.solstitius*, the tuft of setae projecting from the frons is much more prominent than in summer generation *P.glaucus*, where it is greatly reduced (Fig. 4); spring generation *P.glaucus* have similar setation to that of *P.solstitius*. The spring generation of *P.glaucus* can have some *P.canadensis*-like traits (Scriber 1990) that make it more difficult to differentiate from *P.solstitius* based on adult morphology alone. However, throughout much of the range of *P.solstitius*, there is no overlap with the more southern *P.glaucus*. Male genitalic structure is generally regarded as being homogenous among the *glaucus*-complex (Brower 1959; Hagen et al. 1991), but our limited sample suggests that there may be quantitative differences in the number of dorsal tines on the clasper, with *P.canadensis* and *P.solstitius* ranging from one to two spines and *P.glaucus* from one to three (Fig. 9).

Compared to sympatric *P.canadensis* populations, *P.solstitius* can usually be separated with confidence. It is larger with less extensive black markings, most consistently so in the narrower black border of the hindwing anal margin (Fig. 7; Table 1). The narrower margin also results in the large black V (formed by the medial line bridging to the distal part of the anal margin) appearing more U-shaped, versus more sharply V-shaped in *canadensis* (Fig. 7). The ground color is a slightly richer yellow tone. The body vestiture and color differ significantly between the two: the setation of *P.solstitius* is more sparse and shorter, particularly evident on the frons (Fig. 3), the dorsal thorax, and along vein 2A through the black anal margin band of the ventral hindwing (Fig. 7). The head and dorsal thorax are brighter yellow, as is the abdomen. The abdominal subdorsal yellow band is also wider, the male clasper is solid yellow, not interspersed with grey-black scales as in *P.canadensis* (Fig. 8).

Best observed on the underside of the hindwings, the anal margin black band relative to the width of the entire cell containing the band is approximately 10–40% wide in *P.glaucus* and 55–90% wide in *P.canadensis* (Scriber and Ording 2005). The band width averages greater in females than males, but the relative difference between species persists. In *P.solstitius*, this width ranges between approximately 30–55%. Also, on the underside of the hindwings, the lateral interface separating the basal yellow from the black submarginal region is typically somewhat straight in *P.canadensis* (though a common exception being in cell Rs-M_1_ where the line can be bowed inward), noticeably scalloped in *P.glaucus*, with *P.solstitius* demonstrating intermediacy. The hindwing underside submarginal lunules tend toward those of *P.canadensis* in being more rectangular than crescentic.

Comparison of the larval morphology indicates that the color pattern of the first instar is diagnostic for *P.glaucus* and *P.canadensis* (Hagen et al. 1991; Scriber 1998). *Papiliosolstitius* differs from *P.glaucus* and *P.canadensis* in the white dorsal banding pattern (Fig. 10). The prominent white medial saddle, comprised mostly of segments A3-A4, is present in all species. In *P.canadensis*, there are three additional, smaller white bands: two anterior bands formed by white pigmentation on T1 and T3, and a posterior band formed by A8. This banding pattern, with additional anterior-posterior (AP) bands, is consistent in *P.canadensis*. In *P.glaucus*, only the A3-A4 medial saddle is present, and AP bands are absent, the pigmentation on T1, T3, and A8 being dark brown. *Papiliosolstitius* shows intermediacy and variability in the development of the AP bands. Typically, the AP bands are not as prominently white as in *P.canadensis*, but not completely brown as in *P.glaucus*. Development of the AP patterns varies and can be absent (*glaucus*-like) or highly developed (*canadensis*-like), although such variants are rare (< 10% of individuals reared). However, *canadensis*-like larvae never express the same intensity of white pigmentation as that species, although dark variants are essentially undistinguishable from *P.glaucus*. Examples of *glaucus*-like first instars are limited to one field-collection event on a single ash sapling (Kingston, 22.Jul.2023), where five of nine larvae were *glaucus*-like. Clearly, further study of larval variation is needed.

##### ﻿Larval host plants

**Figure 12.**
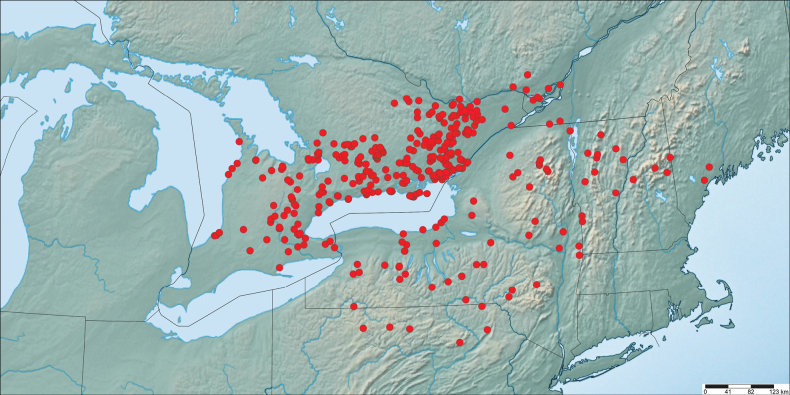
Distribution of examined specimens of *P.solstitius* (voucher data in Suppl. material 1).

In the southern range parts, *Papiliosolstitius* seems to prefer ovipositing on tulip tree (*Liriodendrontulipifera* L.) and hoptree (*Pteleatrifoliata* L.), like *P.glaucus*. Larvae can occur regularly on hoptree where it is planted as an ornamental shrub outside of the natural range (Fig. 12). North of the native ranges of both of these plants (approximately north and east of the region of Toronto, Ontario), *P.solstitius* feeds on *Fraxinuspennsylvanica* and *Prunusserotina*, based on wild-collected ova and larvae and observation of oviposition (Fig. 4b). Larvae demonstrate high survival rates on tulip tree, unlike *P.canadensis*, and also demonstrate survival on quaking aspen (*Populustremuloides* Michaux), unlike *P.glaucus*, but at a rate lower than that of *P.canadensis* (Mercader et al. 2009).

##### ﻿Diapause and phenology

*Papiliosolstitius* exhibits delayed post-diapause pupal development, producing a single summer flight. In Ontario, the flight period commences in late June to early July, peaking in the first half of July (Fig. 2a). Studies on the effect of temperature on pupal development show a similar phenology in New York (Ording et al. 2010). Rearing field-collected ova and larvae from the Kingston region of Ontario further confirm that *P.solstitius* is univoltine with obligate diapause like *P.canadensis*, differing from *P.glaucus* which is facultatively multivoltine (Scriber 2013). Notably, some lab reared pupae overwintered twice, not eclosing until the second year.

Pupae removed from cold storage to a constant temperature of ~23 °C eclosed after 30.4 +/- 5.5 days (male and female combined), or an average of 699 degree-days (DD). *Papiliocanadensis* pupae emerged 19.4 +/- 4.2 days (p < 0.0001), or 446 DD, under the same conditions. In eastern Ontario, accumulated degree-days (above a minimum threshold of 6 °C) for these values correspond to the second week of June (446 DD) and the first week of July (699 DD) (Schmidt and Layberry 2016), precisely when peak emergences of *P.canadensis* and *P.solstitius* are occurring (Fig. 2a). Difference in post-diapause pupal emergence therefore perfectly accounts for the staggered emergence peaks between *P.canadensis* and *P.solstitius* in eastern Ontario. Male and female *P.solstitius* differ in the length of post-diapause development delay. On average, males required approximately 26.6 +/- 3.2 days to eclose compared with 34.2 +/- 4.2 days for females (p = 0.02; *n* = 10; two-tailed T-test). In the wild, this would be expected to translate to a difference in peak flight times between the sexes of approximately 15 days, which matches well with field observations (Fig. 2).

Bivoltine *P.glaucus* populations occur primarily to the south of the range of *P.solstitius*. However, *P.glaucus* is facultatively univoltine or bivoltine at the northern range periphery, contrary to the initial hypothesis that it is unable to switch to univoltinism and limited to regions where it can undergo two annual generations (Hagen et al. 1991). In Ohio and Michigan populations, pupae are induced to enter winter diapause when 4^th^-5^th^ instars experience photoperiods of less than 14 hours (Ryan et al. 2017). Facultative uni- vs. bivoltinism is also demonstrated by our rearing results from the Hamilton, Ontario region, which is north of the bivoltine thermal threshold (Scriber 2013). Lab-reared larvae of spring *P.glaucus* on *L.tulipifera* developed directly into a second generation of adults, despite the rarity of naturally occurring second-flight *P.glaucus* here. Univoltine *P.glaucus* populations probably occur more widely than previously recognized and have added to the complexity of defining the taxa involved in the *glaucus*-complex. Indeed, this could explain the perception of two spring-flying phenotypes (Pavulaan 2024) in regions where both uni- and bivoltine *P.glaucus* occur: offspring developing from either spring-flight (univoltine) or summer-flight (bivoltine) parents experience differing temperature-photoperiod profiles as larvae (known to influence adult phenotype), but both cohorts emerge the following spring. In southern Ontario and the Finger Lakes region of New York, the presence of both spring and summer *P.glaucus* likely accounts for a longer spring abundance peak and a more protracted late summer abundance peak (Fig. 2a, c; see also Schmidt 2020: fig. 7).

##### ﻿Habitat and distribution

Since *Papiliosolstitius*, like its congeners, uses a range of unrelated host plants, it has a similarly broad habitat tolerance for a range of forest, forest edge and woodland habitats. Although habitats of *P.solstitius* and *P.canadensis* overlap widely, the former reaches its highest abundance in or near mesic or moist woodlands, particularly ash-dominated swamps, where ash is common. Conversely, *P.canadensis* is most common in drier upland habitats where trembling aspen is common.

The core range of *Papiliosolstitius* includes eastern and southcentral Ontario, northern and central New York and adjacent Vermont, New Hampshire, and Pennsylvania (Fig. 12), encompassing a minimum land area of approximately 174 000 km^2^ (by comparison, the range extent of *P.appalachiensis* is ~ 140,000 km^2^). In New York, *P.solstitius* inhabits most of the state except the southeast and greater New York City area. In Canada, *P.solstitius* extends from the Montréal, Québec region west to the Bruce Peninsula of Ontario, south to the Niagara region (Fig. 12; Wang 2018; Schmidt 2020; Macnaughton et al. 2020). The western limit appears to be the eastern shores of Lake Huron; we have not seen any verifiable specimens west of there. The *glaucus*-complex has received considerable study in the lower peninsula of Michigan and in Wisconsin, and there is no evidence of delayed flight (July) swallowtails there (Luebke et al. 1988; Stump et al. 2003).

The northern range limit of *P.solstitius* is easily defined since adult morphology and phenology differ considerably from *P.canadensis*. Furthermore, the range limit is climatically constrained since *P.solstitius* larval development is shifted about a month later than *P.canadensis*, and development must be completed before autumnal leaf abscission and frost. The current northern limit is the southern edge of the Algonquin Dome, the lower Ottawa River valley, and the southern edge of the Gatineau/Laurentide escarpment as far east as the Montréal region.

*Papiliosolstitius* has undergone a northward range expansion of several hundred kilometers since the 1970s (Schmidt 2020), as has *P.glaucus* elsewhere (Scriber et al. 2014). In 2022, *P.solstitius* was recorded for the first time near Montebello, Québec. Continuous monitoring at this location since 1994 indicates that *P.solstitius* was not present prior to 2022 (P. Legault, pers. comm). Based on the climatic zones given in Scriber et al. (2014), the distribution of *P.solstitius* approximates the 1300–1400 degree-day (°C) climatic envelope. For context, the northern limit of bivoltine *P.glaucus* is ~1444 DD. The southern (warm) limit of *P.canadensis* appears to be slightly north of this, and is possibly limited by pupal mortality due to prolonged high summer temperatures (Kukal et al. 1991). The northern range limit of *Papiliosolstitius* is likely determined by minimum thermal requirements, given the late seasonal phenology of a July flight period that dictates a shorter window for larval development before autumnal host plant senescence.

The southern range limits of *P.solstitius* are currently difficult to define owing to overlap and confusion with single- and double-brooded *P.glaucus*, and the uncertainty in the northern range limit of *P.glaucus*. Swallowtails that are morphologically consistent with *P.solstitius* and eclosing in the first half of July, when *P.glaucus* is between flights, extend south to approximately 41, 42°N to the eastern seaboard (Fig. 12). In Pennsylvania, the southern extent of *P.solstitius* coincides approximately with the northern limit of *P.glaucus* containing dark morph females (Scriber 1996), extending from Erie to just north of Pittsburgh and east to New York City. It may also extend to the Atlantic coast through Connecticut and Rhode Island based on the phenology information in Pavulaan (2024), but this warrants further study.

The occurrence of *P.canadensis* at the southern range edge, near that of *P.solstitius*, may be more limited than depicted in some range maps (e.g., Pavulaan and Wright 2002; Cech and Tudor 2005; Monroe and Wright 2017). Our examination of putative *P.canadensis* photos from New York and Pennsylvania indicate that most are spring flight *P.glaucus*; CJD has been unable to verify the presence of typical *P.canadensis* in New York state south of the Adirondacks. It is possible and indeed expected that *P.canadensis* is undergoing a northward range contraction with warming climates (Scriber 2013), but this remains unexamined. In the Finger Lakes region of New York, members of the *glaucus*-complex can be observed continuously from mid-May to early September (Fig. 2b). In this region, a pale *canadensis*-like phenotype emerges first, followed by a tiger swallowtail in late May which has historically been referred to as “spring form” *P.glaucus*, and then finally *P.solstitius* in late June to July, and possibly a partial second flight of *P.glaucus* in August (although not all taxa are sympatric everywhere).

##### ﻿Phylogenetic analyses

**Figure 13.**
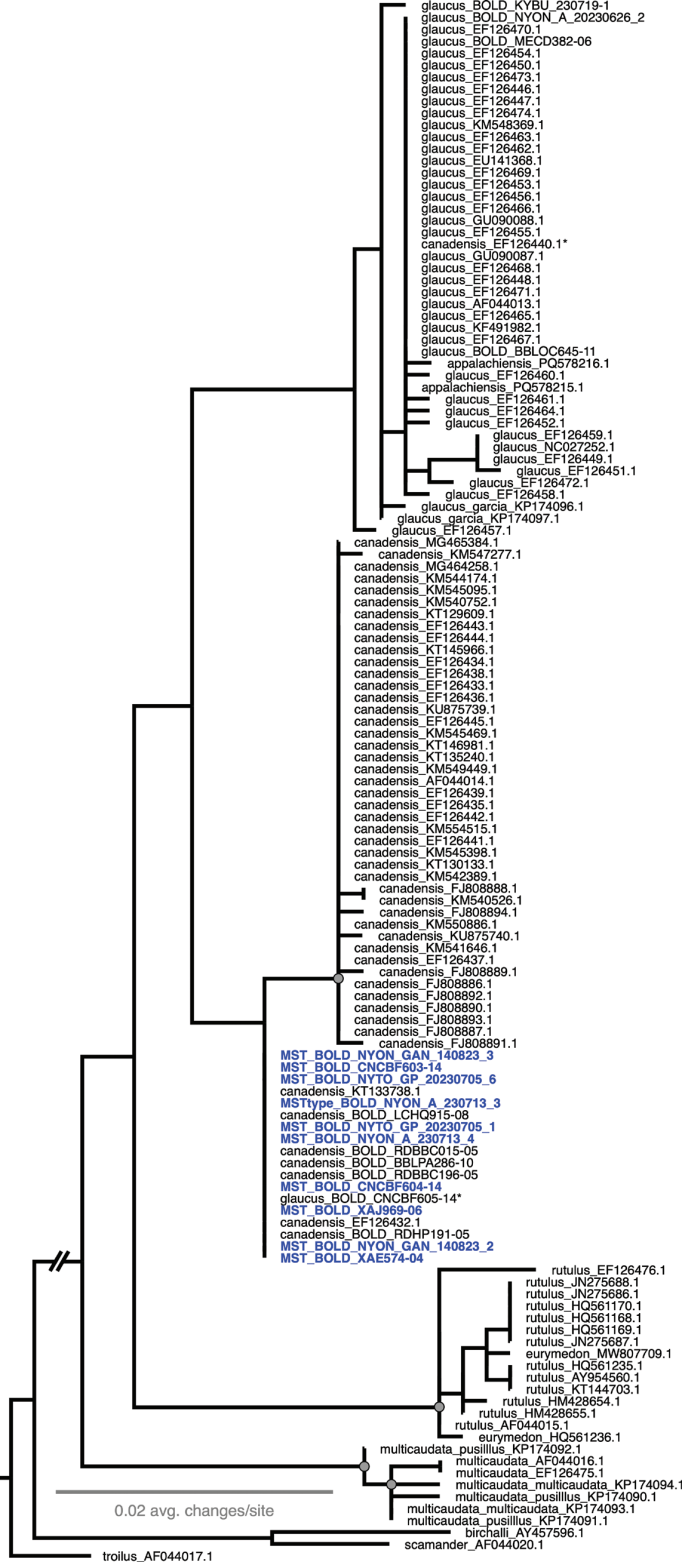
Maximum likelihood tree for COI5. Specimens are labeled with a species epithet determination and NCBI or BOLD accession numbers. *Papiliosolstitius* samples indicated in blue as “MST.” Specimens with asterisks indicate those that fell outside of their typical respective clade. Grey circles indicate strong node support (> 0.95 ufBS and > 0.8 SH-aLRT). All outgroup branch lengths have been edited for space.

**Figure 14.**
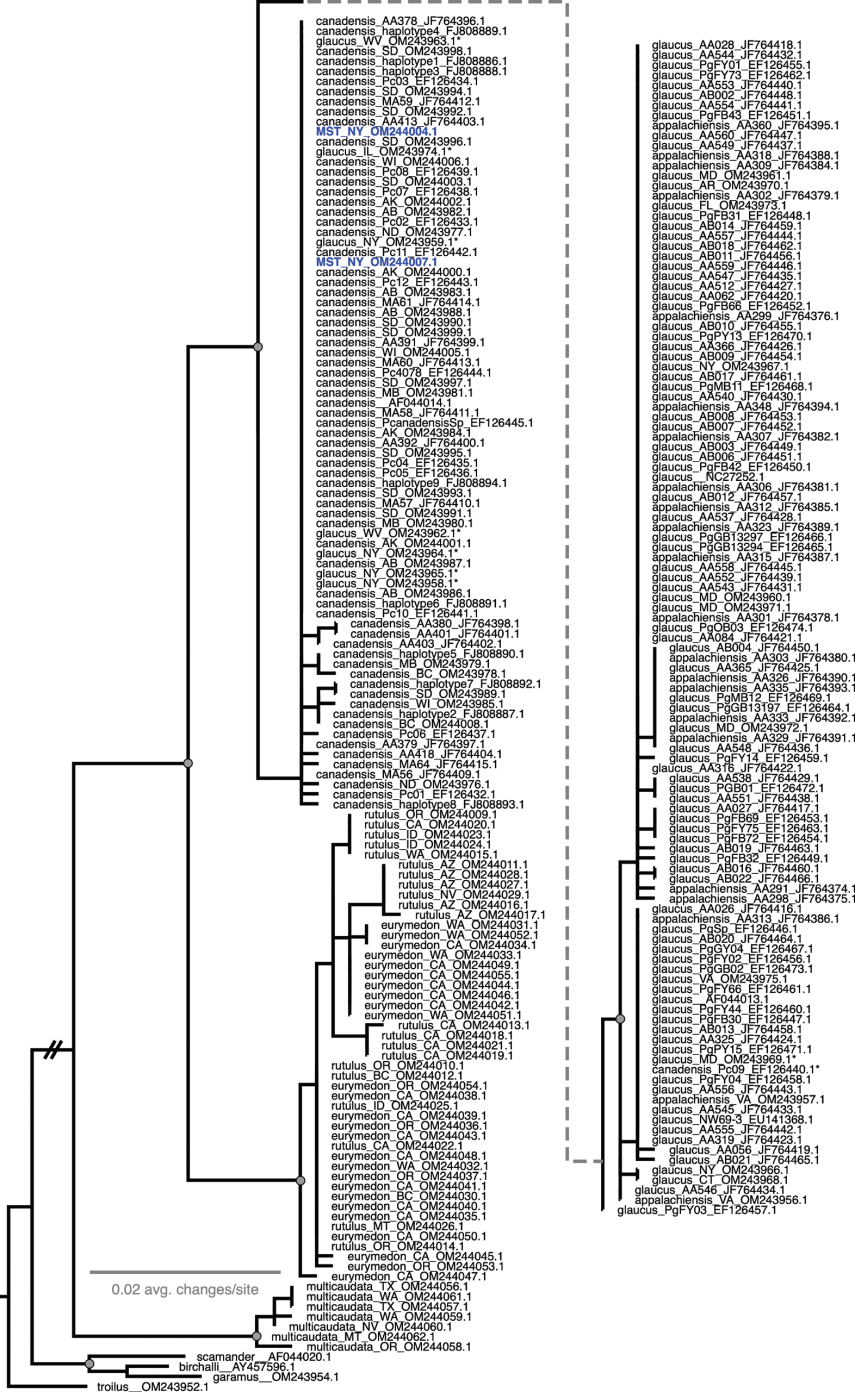
Maximum likelihood tree for COI3. Specimens are labeled with a species epithet determination, state/province, or additional unique identifier, and NCBI accession numbers. *Papiliosolstitius* samples indicated in blue as “MST.” Specimens with asterisks indicate those that fell outside of their typical respective clade. Grey circles indicate strong node support (> 0.95 ufBS and > 0.8 SH-aLRT). All outgroup branch lengths have been edited for space and dotted grey line is a visual link between independently shown parts of the tree.

Both regions of COI recover the same general relationships between members of the *P.glaucus* group, including *P.multicaudata* Kirby, 1884, *P.eurymedon* Lucas, 1852, *P.rutulus* Lucas, 1852, and the *glaucus*-complex clade of *P.glaucus*, *P.canadensis*, and hybrid taxa (*P.appalachiensis*, *P.solstitius*, etc.) (Figs 13, 14). Within the latter, *P.glaucus* and *P.canadensis* almost form reciprocally monophyletic clades in both COI5 and COI3, but in each gene, a handful of specimens fall in the opposing clade (marked with asterisks in Figs 13, 14), and *P.appalachiensis* falls throughout the *P.glaucus* clades in both genes. *Papiliosolstitius* clusters within the *P.canadensis* clade, as does a handful of *P.glaucus*. Notably, there are few nodes with strong branch support within this clade of *P.glaucus*/*P.canadensis*/*P.appalachiensis*/*P.solstitius*, indicating close genetic similarity between all of these entities in their mitochondrial genomes. Excluding specimens with missing data in the 5’ or 3’ ends of their sequences, pairwise sequence identity for haplotypes in this *P.glaucus*/*P.canadensis* clade were > 98% for COI3 and > 97.5% for COI5.

**Figure 15.**
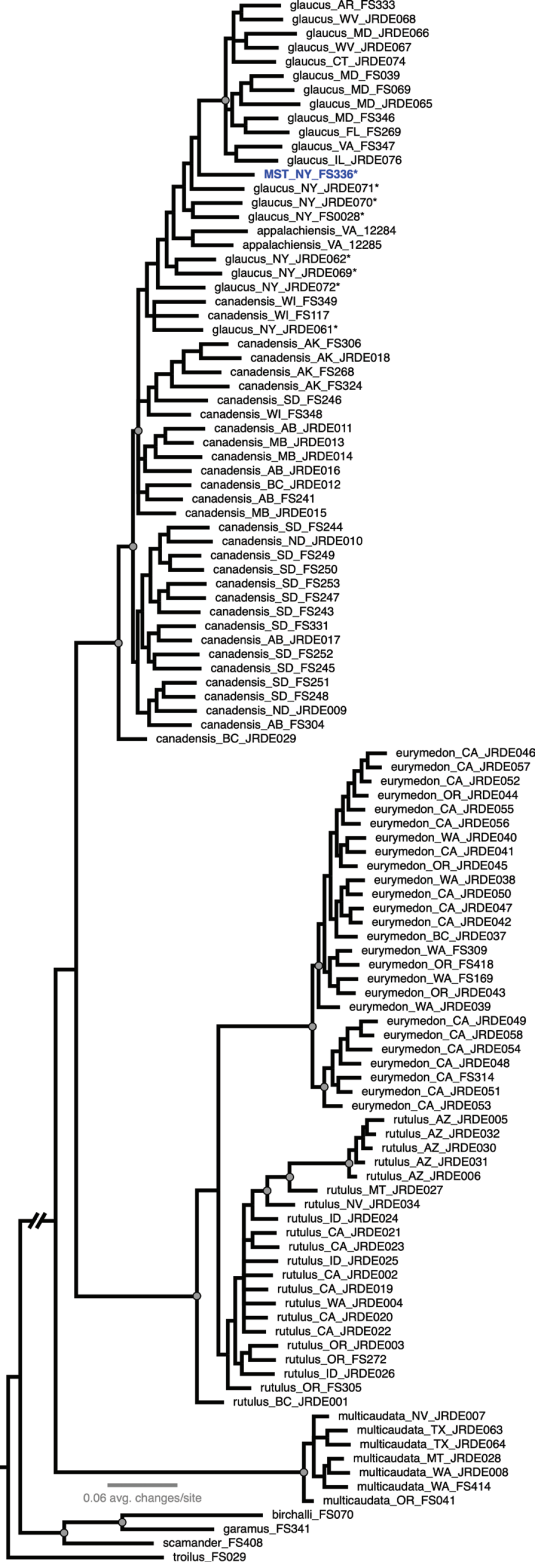
Majority rule consensus tree generated from 3,733 SNPs from Vernygora et al. (2022). Specimens are labeled with a species epithet determination, state/province, and unique identifier, and specimens with asterisks indicate those called “intermediates” in the original publication, including one we identify now as *P.solstitius* (“MST” in blue). Grey circles indicate strong node support (> 0.9 posterior probability support). All outgroup branch lengths have been edited for space.

We re-evaluated identification of specimens sequenced in Vernygora et al. (2022) and conclude that specimens noted as “intermediate” therein are mostly *P.glaucus*, but one is *P.solstitius* (samples annotated with asterisks in Fig. 15). In their SNP-based phylogeny (remade in Fig. 15), these specimens form a paraphyletic grade between typical (and more geographically distant) *P.canadensis* and *P.glaucus*; *Papilioappalachiensis* also falls out in this grade, and as with COI, only a handful of nodes within this broad clade were strongly supported and many of these specimens appeared admixed in Vernygora et al.’s population genetics-oriented analyses.

## ﻿Discussion

Comparison of physiological and morphological traits of the *P.glaucus*-complex taxa in the eastern Great Lakes – northern Appalachian region reveals that the midsummer tiger swallowtail, *Papiliosolstitius* sp. nov., is a distinct, locally common species rather than occasional F1 hybrid individuals between P.glaucus×canadensis. It is geographically widespread over thousands of square kilometers outside of established hybrid zones and is allochronically isolated from its sibling species. Nevertheless, the evolutionary origin of *P.solstitius* through hybridization between *P.glaucus* and *P.canadensis* is likely, as is continued hybridization between the three.

How has a large, conspicuous swallowtail butterfly gone unrecognized in a well-studied region of North America for so long? In hindsight, an earlier study of two univoltine tiger swallowtail populations near Ithaca, NY established the existence of a taxon that was clearly not attributable to either *P.glaucus* or *P.canadensis*, although both entities were referred to as *P.glaucus* (Hagen and Lederhouse 1985). *Papiliocanadensis* was subsequently recognized as a distinct species (Hagen et al. 1991), but the late-flight tiger swallowtails remained a taxonomic enigma and were attributed to a hybrid zone phenomenon (Scriber 1990; Ording et al. 2010; Kunte et al. 2011). *Papiliosolstitius* was documented as early as the 1970s in upstate New York (Hagen and Lederhouse 1985) and eastern Ontario (CNC specimens). However, the earliest literature reference to *P.solstitius* that we could find dates to the mid-1800s from southcentral Ontario. Saunders (1874) noted: “[The tiger swallowtail] appears first on the wing from the middle to the latter end of May, but becomes much more plentiful in July. Whether these July insects are a second brood, or whether the bulk of the chrysalids which have wintered do not mature until about this time we are unable to determine.” As it were, it was not until 1984 that it was proven that the July swallowtails are in fact not a second generation (Hagen and Lederhouse 1985). At the time located near London, Ontario, Saunders’ observations are now easily explained by what would have been either May-flying *P.canadensis* or *P.glaucus* (likely the latter based on current ranges), and *P.solstitius* with its unique July flight time.

Although *P.solstitius* exhibits a mosaic of characters of both *P.glaucus* and *P.canadensis* (Table 1) which might suggest that it is a hybrid, it differs from artificial hybrids in several significant ways (Table 2). Based on the novel detection of late-emerging populations in western Vermont, Ording et al. (2010) suggested *Papiliosolstitius* to be of very recent hybrid origin mediated by climatic amelioration. The historic documentation and large geographic range, much of it beyond the contact zone between *P.glaucus* and *P.canadensis*, counter this hypothesis. Notably, the delayed pupal emergence with a single summer flight differs from lab hybrids which emerge in the spring (Ording et al. 2010). Our data for post-diapause pupal development of Ontario *P.solstitius* are comparable to values given by Ording et al. (2010) from Vermont populations: under controlled laboratory conditions, post-diapause pupae of *Papiliosolstitius* and *P.canadensis* emerged after an average of 828 DD and 450 DD, respectively (Ording et al. 2010), versus our results for Ontario populations of both species at 690 DD and 437 DD. In eastern Ontario, the average peak flight period of *Papiliosolstitius* is 11–20 July, compared to 1–10 June for *P.canadensis* (Fig. 2a). *Papiliosolstitius* is distinct from artificial F1 hybrids and both parental species in this regard, which emerge in the spring (Ording et al. 2010). Importantly, this difference results in temporal reproductive isolation between *P.solstitius* and *P.canadensis*/*glaucus*. Within the *P.glaucus* group, delayed pupal emergence is unique to *P.solstitius*, and understanding the adaptive significance of this may provide key insights into its evolutionary history. Possibly it is a mechanism to escape pupal mortality due to summer heat, to which *P.canadensis* is susceptible (Kukal et al. 1991).

**Table 2.** Comparison of genetic and ecological traits among species of the *Papilioglaucus*-complex. Sourced from Kunte et al. (2011), Scriber and Ording (2005), and this paper. The recently described *P.bjorkae* is excluded because most traits remain undefined or unknown (see Introduction).

Trait	*P.glaucus*	*P.appalachiensis*	*P.solstitius* sp. nov.	*P.canadensis*	*F1* lab hybrid
Thermal habitat	warm	intermediate	intermediate	cool	na
Pupal diapause	facultative	obligatory	obligatory	obligatory	Z-linked
Voltinism	bivoltine	univoltine	univoltine	univoltine	photoperiod (Z)
Larval survival: aspen	low	high	high	high	high
Larval survival: tuliptree	high	high	high	low	high
Body size	large	large	intermediate	small	intermediate
Female polymorphism	mimetic	mimetic	non-mimetic	non-mimetic	W-linked
Pupal emergence	early	early	delayed	early	heterozygous (Z)
Flight season	early + late	early	mid	early	n.a.
mtDNA	*glaucus*-like	*glaucus*-like	*canadensis*-like	*canadensis*-like	maternal
Z: Kettin	*glaucus*	*canadensis*	*canadensis*	heterozygous	heterozygous (Z)
Z: TH	*glaucus*	*canadensis*	*canadensis*	*canadensis*	heterozygous (Z)
Z: Tpi	*glaucus*	*canadensis*	*canadensis*	*canadensis*	heterozygous (Z)
Z: Period	*glaucus*	*canadensis*	*canadensis*	*canadensis*	heterozygous (Z)
Z: PAH	*glaucus*	*canadensis*	*canadensis*	*canadensis*	heterozygous (Z)
LDH allozyme	100	80 / 40	80 / 40	80 / 40	heterozygous (Z)
LDH20 “hybrizyme”	-	+	+	-	n.a.
PGD allozyme	100 / 50	100 / 50	100/50 (40-50%)	125/80/150	heterozygous (Z)

Hybridization between *P.glaucus* and *P.canadensis* has been well-documented using molecular and morphological evidence, and only some purported hybrid populations can be attributed to *Papiliosolstitius*. The most extensively studied hybrid zone between *P.glaucus* and *P.canadensis* is a narrow geographic zone across Michigan’s lower peninsula and into Wisconsin (Luebke et al. 1988; Hagen et al. 1991). Here, the hybrid zone is dictated by a narrow band of the thermal landscape that limits the occurrence of *P.canadensis* to the north and *P.glaucus* to the south. There is no evidence that *Papiliosolstitius* occurs this far west. To the east, the biogeography of the *P.glaucus* group is more difficult to untangle, influenced by the complex topography of the northern Appalachians, Frontenac Arch, Alleghany Plateau, and Adirondack Mtns with the added complexity of Great Lakes weather effects. Unlike the region west of Lake Michigan, large gaps occur between the ranges of *P.glaucus* and *P.canadensis* here, but there is undoubtedly ongoing gene flow between *P.solstitius* and its sibling species and is fertile ground for future molecular study.

Some of the initial genetic work on the *glaucus*-complex included samples of *P.solstitius* and indicated different allele frequencies of alpha-galactosaminidase compared to *P.canadensis* (Hagen and Lederhouse 1985). *Papiliosolstitius* also possesses a unique allozyme, LDH-20, not present in other *P.glaucus* group species (Scriber and Ording 2005). The presence of molecular traits unique to *P.solstitius* not known from either putative parent species cannot easily be explained by ongoing hybridization.

Considering recent genetic data together, it is clear that the standard barcoding gene, COI, is unable to confidently separate *P.solstitius* from *P.canadensis*. The handful of specimens falling outside their respective clades for COI may be indicative of geographic variation that has been historically unsampled/unsequenced, or more varied hybrid interactions between *P.glaucus* and *P.canadensis*. Ignoring these specimens that fall outside of their respective clades, *P.solstitius* clearly has more *P.canadensis* maternal influence, but its nuclear genome is less clear as our sampling is more limited and shows a paraphyletic grade in phylogenetic analyses (Fig. 15) and varied signals of admixture in the results of Vernygora et al. (2022). More comprehensive population genomic sampling will be required to tease apart the genetic situation of *P.solstitius*, *P.appalachiensis* (Cong et al. 2015), and the other hybrids/entities documented in this species group (Ryan et al. 2016, 2017, 2018; Pavulaan 2024). Although Scriber and Ording (2005) and Kunte et al. (2011) potentially addressed *P.solstitius* with other putative hybrids within the *P.glaucus* group, modern genomic methods should be used to properly characterize population-wide genetic variation throughout this broad geographic region and other hybrid entities within the *P.glaucus* group (Ryan et al. 2016).

Current evidence is consistent with the possibility that *P.solstitius* has a recombinant evolutionary origin similar to that of *P.appalachiensis*. However, most questions regarding the evolutionary origin of this taxon, and its role within the speciation of the *P.glaucus*-complex, remain to be answered. It is our hope that recognizing and defining the taxonomic identity of this unique evolutionary lineage provides a staging point in the fertile grounds for future research.

## Supplementary Material

XML Treatment for Papilio solstitius

## ﻿Additional information

### Author ORCIDs

B. Christian Schmidt https://orcid.org/0000-0003-4160-7629

### Data availability

All of the data that support the findings of this study are available in the main text or Supplementary Information.

## Supplementary materials

Supplementary material 1 Voucher data for examined specimens of *Papiliosolstitius* Data type xlsx https://binary.pensoft.net/file/1251231This dataset is made available under the Open Database License (http://opendatacommons.org/licenses/odbl/1.0/). The Open Database License (ODbL) is a license agreement intended to allow users to freely share, modify, and use this Dataset while maintaining this same freedom for others, provided that the original source and author(s) are credited.Charles J. DeRoller, Xi Wang, Julian R. Dupuis, B. Christian Schmidt

## References

[B1] Brower LP (1959) Speciation in butterflies of the *Papilioglaucus* group. I. Morphological relationships and hybridization. Evolution 13(1): 40–63. 10.2307/2405944

[B2] Cech R Tudor G (2005) Butterflies of the East Coast: an observer’s guide. Princeton University Press, Princeton, New Jersey, 234 pp. 10.1515/9780691261164

[B3] Condamine FL Allio R Reboud EL Dupuis JR Toussaint EFA Mazet N Hu SJ Lewis DS Kunte K Cotton AM Sperling FAH (2023) A comprehensive phylogeny and revised taxonomy illuminate the origin and diversification of the global radiation of *Papilio* (Lepidoptera: Papilionidae). Molecular Phylogenetics and Evolution 183: 107758. 10.1016/j.ympev.2023.107758 36907224

[B4] Cong Q Borek D Otwinowski Z Grishin NV (2015) Tiger swallowtail genome reveals mechanisms for speciation and caterpillar chemical defense. Cell Reports 10(6): 910–919. 10.1016/j.celrep.2015.01.026 25683714 PMC8935626

[B5] Edgar RC (2004) MUSCLE: multiple sequence alignment with high accuracy and high throughput. Nucleic Acids Research 32(5): 1792–1797. 10.1093/nar/gkh340 15034147 PMC390337

[B6] Folmer O Black M Hoeh W Lutz R Vrijenhoek R (1994) DNA primers for amplification of mitochondrial cytochrome c oxidase subunit I from diverse metazoan invertebrates. Molecular Marine Biology and Biotechnology 3(5): 294–299. 7881515

[B7] Guindon S Dufayard JF Lefort V Anisimova M Hordijk W Gascuel O (2010) New algorithms and methods to estimate maximum-likelihood phylogenies: assessing the performance of PhyML 3.0. Systematic Biology 59(3): 307–321. 10.1093/sysbio/syq010 20525638

[B8] Hagen RH Lederhouse RC (1985) Polymodal emergence of the tiger swallowtail, *Papilioglaucus* (Lepidoptera: Papilionidae): source of a false second generation in central New York State. Ecological Entomology 10(1): 19–28. 10.1111/j.1365-2311.1985.tb00531.x

[B9] Hagen RH Lederhouse RC Bossart JL Scriber JM (1991) *Papiliocanadensis* and *P.glaucus* (Papilionidae) are distinct species. Journal of the Lepidopterists’ Society 45(4): 245–258.

[B10] Hall PW Jones C Guidotti A Hubley B (2014) The ROM field guide to butterflies of Ontario. Royal Ontario Museum, Toronto, Ontario, 488 pp.

[B11] Hebert PD Cywinska A Ball SL deWaard JR (2003) Biological identifications through DNA barcodes. Proceedings of the Royal Society B: Biological Sciences 270(1512): 313–321. 10.1098/rspb.2002.2218 PMC1691236 12614582

[B12] Hoang DT Chernomor O von Haeseler A Minh BQ Vinh LS (2018) UFBoot2: improving the ultrafast bootstrap approximation. Molecular Biology and Evolution 35(2): 518–522. 10.1093/molbev/msx281 29077904 PMC5850222

[B13] Kalyaanamoorthy S Minh BQ Wong TKF von Haeseler A Jermiin LS (2017) ModelFinder: fast model selection for accurate phylogenetic estimates. Nature Methods 14(6): 587–589. 10.1038/nmeth.4285 28481363 PMC5453245

[B14] Kukal O Ayres MP Scriber JM (1991) Cold tolerance of the pupae in relation to the distribution of swallowtail butterflies. Canadian Journal of Zoology 69(12): 3028–3037. 10.1139/z91-427

[B15] Kunte K Shea C Aardema ML Scriber JM Juenger TE Gilbert LE Kronforst MR (2011) Sex chromosome mosaicism and hybrid speciation among tiger swallowtail butterflies. PLOS Genetics 7(9): e1002274. 10.1371/journal.pgen.1002274 PMC3169544 21931567

[B16] Larsson A (2014) AliView: a fast and lightweight alignment viewer and editor for large data sets. Bioinformatics 30(22): 3276–3278. 10.1093/bioinformatics/btu531 25095880 PMC4221126

[B17] Layberry RA Hall PW Lafontaine JD (1998) The Butterflies of Canada. Toronto: University of Toronto Press, Toronto, Ontario, 354 pp. 10.3138/9781442623163

[B18] Linnaeus C (1758) Systema Naturae, 10^th^ edn., Vol. 1, Stockholm, 824 pp.

[B19] Luebke HJ Scriber JM Yandell BS (1988) Use of multivariate discriminant analysis of male wing morphometrics to delineate a hybrid zone for *Papilioglaucusglaucus* and *P.g.canadensis* in Wisconsin. The American Midland Naturalist 119(2): 366–379. 10.2307/2425819

[B20] Macnaughton A Layberry R Cavasin R Edwards B Jones C (2020) Ontario butterfly atlas. https://www.ontarioinsects.org/atlas/

[B21] Mercader RJ Aardema ML Scriber JM (2009) Hybridization leads to host-use divergence in a polyphagous butterfly sibling species pair. Oecologia 158(4): 651–662. 10.1007/s00442-008-1177-9 18949489

[B22] Monroe JL Wright DM (2017) . Butterflies of Pennsylvania: a field guide. University of Pittsburgh Press, Pittsburgh, Pennsulvania, 336 pp.

[B23] Nguyen LT Schmidt HA von Haeseler A Minh BQ (2015) IQ-TREE: a fast and effective stochastic algorithm for estimating maximum-likelihood phylogenies. Molecular Biology and Evolution 32(1): 268–274. 10.1093/molbev/msu300 25371430 PMC4271533

[B24] Ording GJ Mercader R Aardema ML (2010) Allochronic isolation and incipient hybrid speciation in tiger swallowtail butterflies. Oecologia 162(2): 523–531. 10.1007/s00442-009-1493-8 19937057

[B25] Pavulaan H (2024) Determination of a new spring-flying species of the *Pterourusglaucus* complex (Papilionidae) in southern New England. The Taxonomic Report of the International Lepidoptera Survey 12(1): 1–26. 10.5281/zenodo.13952895

[B26] Pavulaan H Wright D (2002) *Pterourusappalachiensis* (Papilionidae: Papilioninae), a new swallowtail butterfly from the Appalachian Region of the United States. The Taxonomic Report of the International Lepidoptera Survey 3(7): 1–20.

[B27] Pelham JP Pohl GR (2023) Family Papilionidae Latreille, [1802] (apollos and swallowtails). In: Pohl GR Nanz SR (Eds.) Annotated taxonomic checklist of the Lepidoptera of North America, North of Mexico.Wedge Entomological Research Foundation, Bakersfield, California, 193–196.

[B28] Rambaut A Drummond AJ (2010) FigTree v1.4.4. Institute of Evolutionary Biology, University of Edinburgh. http://www.treebioedacuk/software/figtree

[B29] Ridgway R (1912) . Color Standards and Color Nomenclature. Washington, DC. 10.5962/bhl.title.144788

[B30] Ronquist F Teslenko M Van Der Mark P Ayres DL Darling A Höhna S Larget B Liu L Suchard MA Huelsenbeck JP (2012) MrBayes 3.2: efficient Bayesian phylogenetic inference and model choice across a large model space. Systematic biology 61(3): 539–542. 10.1093/sysbio/sys029 22357727 PMC3329765

[B31] Rothschild W Jordan K (1906) A revision of the American papilios. Novitates Zoologicae 13: 411–744. 10.5962/bhl.part.22801

[B32] Ryan SF Valella P Thivierge G Aardema ML Scriber JM (2016) The role of latitudinal, genetic and temperature variation in the induction of diapause of *Papilioglaucus* (Lepidoptera: Papilionidae). Insect Science 25(2): 328–336. 10.1111/1744-7917.12423 27900827

[B33] Ryan SF Fontaine MC Scriber JM Pfrender ME O’Neil ST Hellmann JJ (2017) Patterns of divergence across the geographic and genomic landscape of a butterfly hybrid zone associated with a climatic gradient. Molecular Ecology 26(18): 4725–4742. 10.1111/mec.14236 28727195

[B34] Ryan SF Deines JM Scriber JM Pfrender ME Jones SE Emrich SJ Hellmann JJ (2018) Climate-mediated hybrid zone movement revealed with genomics, museum collection, and simulation modeling. Proceedings of the National Academy of Sciences of the United States of America 115(10): E2284–E2291. 10.1073/pnas.1714950115 PMC5877999 29463695

[B35] Saunders WH (1874) On some of our common insects. II. The tiger swallow tail – *Papilioturnus*, L. The Canadian Entomologist 6(1): 2–5. 10.4039/Ent62-1

[B36] Schmidt BC (2018) Cryptic species among bumblebee mimics: an unrecognized *Hemaris* hawkmoth (Lepidoptera: Sphingidae) in eastern North America. Zootaxa 4399(1): 32–48. 10.11646/zootaxa.4399.1.2 29690328

[B37] Schmidt C (2020) More on Ontario tiger swallowtails. In: Hockley L Macnaughton A (Eds) Ontario Lepidoptera 2019.Toronto Entomologists’ Association Occasional Publication #51-2020, Toronto, Ontario, 3–11. https://www.ontarioinsects.org/publications/Summaries/2019.pdf

[B38] Schmidt BC Layberry RA (2016) What Azure blues occur in Canada? A re-assessment of *Celastrina* Tutt species (Lepidoptera, Lycaenidae). ZooKeys 26(584): 135–164. 10.3897/zookeys.584.7882 PMC4857028 27199600

[B39] Scriber JM (1990) Interaction of introgression from *Papilioglaucuscanadensis* and diapause in producing “spring form” eastern tiger swallowtail butterflies, *P.glaucus* (Lepidoptera: Papilionidae). The Great Lakes Entomologist 23(3): 127–138.

[B40] Scriber JM (1996) Tiger tales: natural history of native North American swallowtails. American Entomologist 42(1): 19–32. 10.1093/ae/42.1.19

[B41] Scriber JM (1998) The inheritance of diagnostic larval traits for interspecific hybrids of *Papiliocanadensis* and *P.glaucus* (Lepidoptera: Papilionidae). The Great Lakes Entomologist 31(2): 113–124. 10.22543/0090-0222.1955

[B42] Scriber JM (2010) Impacts of climate warming on hybrid zone movement: Geographically diffuse and biologically porous “species borders.” Insect Science 18(2): 121–159. 10.1111/j.1744-7917.2010.01367.x

[B43] Scriber JM (2013) Climate-driven reshuffling of species and genes: potential conservation roles for species translocations and recombinant hybrid genotypes. Insects 5(1): 1–61. 10.3390/insects5010001 26462579 PMC4592632

[B44] Scriber JM Ording GJ (2005) Ecological speciation without host plant specialization; possible origins of a recently described cryptic *Papilio* species. Entomologia Experimentalis et Applicata 115(1): 247–263. 10.1111/j.1570-7458.2005.00285.x

[B45] Scriber JM Elliot B Maher E McGuire M Niblack M (2014) Adaptations to “thermal time” constraints in *Papilio*: latitudinal and local size clines differ in response to regional climate change. Insects 5(1): 199–226. 10.3390/insects5010199 26462585 PMC4592633

[B46] Shapiro AM (1974) The butterflies and skippers of New York (Lepidoptera: Papilionoidea, Hesperioidea). Search 4(3): 1–60.

[B47] Sperling FAH (1993) Mitochondrial DNA variation and Haldane’s rule in the *Papilioglaucus* and *P.troilus* species groups. Heredity 71(3): 227–233. https://www.nature.com/articles/hdy1993130

[B48] Stump AD Sperling FA Crim A Scriber JM (2003) Gene flow between Great Lakes region populations of the Canadian tiger swallowtail butterfly, *Papiliocanadensis*, near the hybrid zone with *P.glaucus* (Lepidoptera: Papilionidae). The Great Lakes Entomologist 36: 41–52. https://scholar.valpo.edu/tgle/vol36/iss1/8/

[B49] Vernygora OV Campbell EO Grishin NV Sperling FAH Dupuis JR (2022) Gauging ages of tiger swallowtail butterflies using alternate SNP analyses. Molecular Phylogenetics and Evolution 171: 107465. 10.1016/j.ympev.2022.107465 35351633

[B50] Wang X (2018) An update on tiger swallowtails in Ontario. In: Cavasin R Linton JE (Eds) Ontario Lepidoptera 2017.Toronto Entomologists’ Association Occasional Publication #48-2018, Toronto, Ontario, 25–29. https://www.ontarioinsects.org/publications/Summaries/2017_tigers.pdf

[B51] Zhang W Kunte K Kronforst MR (2013) Genome-wide characterization of adaptation and speciation in tiger swallowtail butterflies using de novo transcriptome assemblies. Genome Biology and Evolution 5(6): 1233–1245. 10.1093/gbe/evt090 23737327 PMC3698933

